# A Robust Method for Validating Orientation Sensors Using a Robot Arm as a High-Precision Reference

**DOI:** 10.3390/s24248179

**Published:** 2024-12-21

**Authors:** József Kuti, Tamás Piricz, Péter Galambos

**Affiliations:** Antal Bejczy Center for Intelligent Robotics, Obuda University, 1034 Budapest, Hungary; tamas.piricz@irob.uni-obuda.hu

**Keywords:** orientation sensing, inertial measurement unit, MEMS, gyroscope, VR headset, robotics

## Abstract

This paper presents a robust and efficient method for validating the accuracy of orientation sensors commonly used in practical applications, leveraging measurements from a commercial robotic manipulator as a high-precision reference. The key concept lies in determining the rotational transformations between the robot’s base frame and the sensor’s reference, as well as between the TCP (Tool Center Point) frame and the sensor frame, without requiring precise alignment. Key advantages of the proposed method include its independence from the exact measurement of rotations between the reference instrumentation and the sensor, systematic testing capabilities, and the ability to produce repeatable excitation patterns under controlled conditions. This approach enables automated, high-precision, and comparative evaluation of various orientation sensing devices in a reproducible manner. Moreover, it facilitates efficient calibration and analysis of sensor errors, such as drift, noise, and response delays under various motion conditions. The method’s effectiveness is demonstrated through experimental validation of an Inertial Navigation System module and the SLAM-IMU fusion capabilities of the HTC VIVE VR headset, highlighting its versatility and reliability in addressing the challenges associated with orientation sensor validation.

## 1. Introduction

Orientation sensors, whether standalone or integrated into pose-tracking systems, are extensively used in drones, mobile robots, medical applications [1], and immersive visualization devices [2]. These sensors provide absolute measurements, differential rotations, or a combination of both. For instance, Inertial Navigation Systems integrate data from a gyroscope, accelerometer, and magnetometer, while 3D SLAM applications can leverage IMU data alongside odometry for precise localization and mapping.

Sensor systems with stable external references like motion capture cameras, VR tracking devices, and electromagnetic trackers provide reliable absolute orientation with moderate noise levels. However, the increasingly popular MEMS-based and the so-called inside-out vision-based systems are less robust against noise, bias, drift, and delay. For example, concerning MEMS sensors, Shi et al. pointed out that environmental factors, particularly ambient temperature, significantly affect the bias and drift of these sensors, which can lead to decreased orientation estimation accuracy [3]. Another analysis by Suvorkin et al. utilized the Allan deviation slope method to evaluate noise characteristics across different grades of sensors. This analysis provides insights into the stability and reliability of IMU measurements over time, which is essential for applications requiring high precision [4]. Understanding noise characteristics is crucial for interpreting IMU data accurately and ensuring its validity in practical applications. With the emergence of more advanced observer-based and dynamic filtering techniques for enhancing raw IMU data, such as those proposed in [5,6], the need for repeatable and automated validation methods has become increasingly important.

Inside-out tracking systems, such as those used in Microsoft’s HoloLens or HTC Vive, rely on onboard cameras to track the environment and determine orientation. While these systems offer the advantage of not requiring external infrastructure, they are highly sensitive to environmental conditions, such as lighting and occlusions. A study by Zhang et al. found that inside-out tracking systems could experience substantial errors in orientation estimation when subjected to rapid movements or changes in lighting conditions [7]. Additionally, the latency introduced by image processing can lead to noticeable delays in orientation updates, impacting user experience. Similarly, a paper by Niehorster et al. on the HTC Vive’s inside-out tracking system highlighted that while the precision of tracking measurements is high, the system can experience significant offsets when tracking is temporarily lost, affecting overall accuracy [8]. Other studies using the HTV Vive tracker give similar conclusions [9,10]. This illustrates the challenges faced by systems that rely on internal measurements compared with those that utilize stable external references.

These imperfections underline the importance of validation methodologies that allow for precise and automatic comparisons of various sensors under multiple conditions in a repeatable way. The validation of orientation sensors relies heavily on the establishment of ground truth data. Ground truth serves as a reference against which the performance of the sensors can be assessed. Various methods for obtaining ground truth have been documented in the literature, each with its unique advantages and applications.

One of the most reliable forms of ground truth for validating IMU orientation sensors is the use of motion capture systems. These systems provide high-precision data regarding the position and orientation of subjects in three-dimensional space. For instance, Morrow et al. conducted a validation study where they compared the kinematic data obtained from a commercially available IMU system against a standard optical motion capture system [11]. Inside-out tracking systems have been validated in similar setups [9,12]. This comparative approach is widely recognized for its effectiveness if a motion capture laboratory is available.

Another approach to obtaining reliable references is the use of mechanical fixtures. The use of fixtures in experimental setups allows for controlled conditions that can significantly enhance the reliability of sensor validation processes. Bliley et al. [13] described a validation method for wearable motion-sensing devices that involved a mechanical fixture. Their approach utilized a platform rotated by a servo motor, which allowed for precise control over the orientation of the sensors being tested. This method ensured that the sensors were subjected to known and repeatable movements, facilitating accurate comparisons between the sensor outputs and the expected values. Additionally, Eastman et al. [14] highlighted the importance of using fixtures in their work on 6DoF (six degrees of freedom) object pose ground truth measurements. They employed an aluminum pose fixture along with a laser tracker to establish a reliable reference for validating sensor outputs. This combination of a rigid fixture and precise measurement tools allowed for high accuracy in determining the orientation and position of objects. Similarly, Herickhoff et al. [15] utilized a lightweight plastic fixture to hold an IMU sensor. The adaptability of such fixtures makes them valuable tools in various applications, including medical imaging and motion analysis.

These published methods lack the ability to provide a programmatic way to generate test motions and then analyze the results in a tractable manner. Using a robot arm as a ground truth reference would provide important benefits: the robot arm can provide precise and repeatable movements, allowing for accurate comparisons between the sensor outputs and the known positions and orientations of the arm. This motivation has been supported by studies from the literature in the field. Schmidt and Kraft noted that accurate ground truth trajectories can be obtained when a camera is mounted on an industrial robot arm, which allows for reliable evaluations of visual SLAM (Simultaneous Localization and Mapping) algorithms [16]. Several papers, such as [17,18,19], highlight the advantages of using robot arms to create motion patterns for testing IMUs. A notable method proposed by Hoslip et al. employs an industrial robot to evaluate the performance of multiple IMU sensors simultaneously [20]. However, despite the use of various robotic manipulators, no systematic approach has been published to mathematically address the geometric relationships between the robot’s reference frame and the sensor’s reference frame.

Authors faced this unsolved issue during the experimental validation of a generic direction and orientation tracking method based on an IMU sensor, which has been published in [21]. In our former work, we proposed a mathematical framework for the calibration and tracking of objects with one functional direction (continuous rotational symmetric case) and multiple functional directions (non-symmetric case). This previous work has inspired the current study which proposes a method for the validation of orientation sensors with respect to an external reference. The proposed method is agnostic to the external (reference) measurement system, but it is discussed considering a robotic arm as a robust integrated actuation and sensing device, and the robotics terminology is used throughout the discussion.

**Figure 1:**
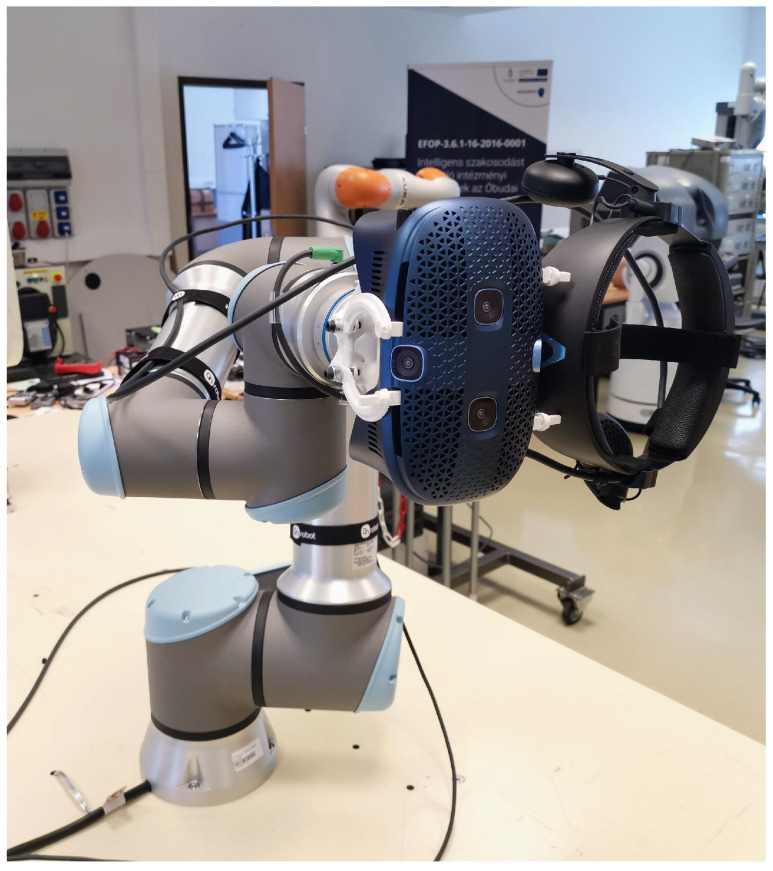
HTC Vive Cosmos VR headset mounted on a UR16e robot.

In our setup, a commercial robot arm is employed for validation, providing a precise orientation reference and enabling automated testing capabilities (Figure 1). This approach not only ensures high accuracy but also facilitates systematic and repeatable validation processes.

**Figure 2:**
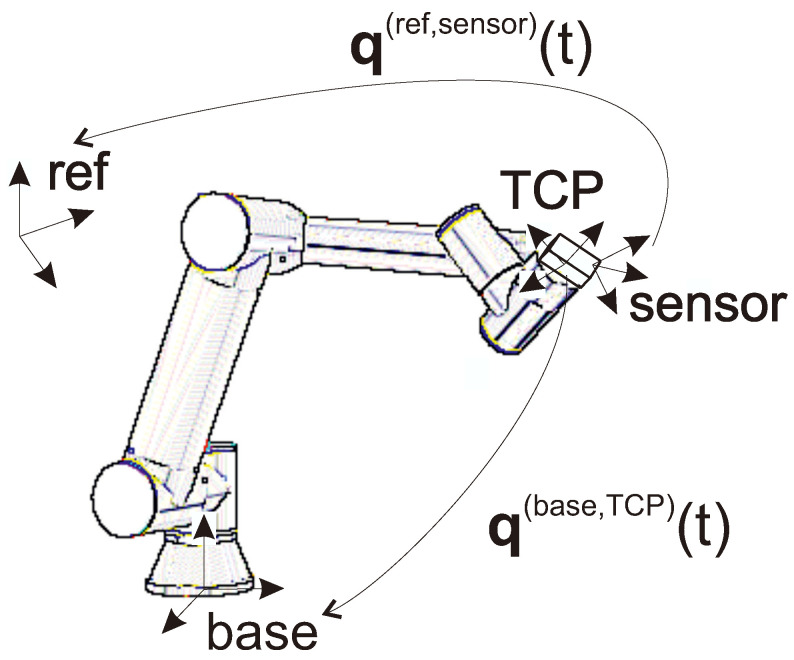
Robot manipulator with an orientation sensor (illustrated as a box on the flange) with the frames and measured quantities considered throughout this paper.

The validation of orientation sensors using any test setup introduces the challenge of dealing with different reference and target frames. The sensor initializes its own unknown reference frame and measures the orientation of a frame fixed to the sensor relative to this reference. The analogous frames of an industrial manipulator are called base frame and TCP frame, respectively (see Figure 2). We can suppose that the industrial robot’s internal measurement system provides reliable position and accurate orientation information regarding the base to TCP transformation [22,23].

As its main contribution, this paper proposes a tractable method to approximate the rotation transformation between the robot’s base frame and the sensor frame, as well as between the TCP (Tool Center Point) frame and the sensor frame. The method involves two main steps: first, deriving an initial estimate using geometric principles, followed by a local optimization to minimize the impact of measurement noise and errors. The proposed method relies solely on easily obtainable logged data of the robot’s TCP orientations and the sensor’s outputs, enabling automated and repeatable testing under a wide range of operational conditions and motion dynamics.

To demonstrate the method’s effectiveness, experiments were conducted using a UR16e robot [24] with two distinct sensors: the widely used ICM20948 IMU sensor [25] and the HTC VIVE headset’s inside-out tracking system [8,26,27,28].

This paper is organized as follows: Section 2 defines the problem and introduces the relevant notations. Section 3 elaborates on the proposed calibration method. Section 4 presents experimental results obtained with various sensors and provides a comparative analysis. Section 5 outlines future research activities and potential practical applications. Finally, Section 6 summarizes the findings. Appendix A includes details on vector and quaternion operations, along with their respective notations, as used throughout the paper. Original measurement data associated with the content of this paper can be found at [29].

## 2. Problem Description

In the investigated measurement setup, the $q^{\left(base,TCP\right)}\left(t\right)$ orientation can be obtained from the pose of the manipulator, while the sensor provides its $q^{\left(ref,sensor\right)}\left(t\right)$ orientation with respect to its own reference frame; see Figure 2.

Note that the orientation sensor can be attached to the robot’s end-effector in any arbitrary relative orientation. There are no restrictions on this relative orientation, as it does not affect the calibration accuracy. However, it is essential that the relative orientation remains unchanged throughout the entire measurement process.

In an ideal case, there exist $q^{\left(base,ref\right)}$ and $q^{\left(TCP,sensor\right)}$ orientations such that

(1) $$q^{\left(base,TCP\right)}\left(t\right)\cdot q^{\left(TCP,sensor\right)}=q^{\left(base,ref\right)}\cdot q^{\left(ref,sensor\right)}\left(t\right)$$

for all $t\in [0,t_{meas}]$. However, in practical scenarios, there is an error resulting from the measurement noises and the drift of the sensor.

In the following, the $q^{\left(base,TCP\right)}\left(t\right)$ value (read from the robot controller) will be considered as ground truth. The computation of $q^{\left(TCP,sensor\right)}$ and $q^{\left(base,ref\right)}$ orientations from some initial data will be referred to as calibration.

Considering the whole recorded dataset, the angle error of Equation (1) will be analyzed to obtain the measurement noise and the drift of the sensor.

## 3. Calibration Method

The calibration is performed using solely the first $0\leq t<t_{cal}$ values of the measured data. This approach enables the analysis of sensor drift in the subsequent measurements for $t\geq t_{cal}$.

The method requires at least three values from the $t<t_{cal}$ portion of the measured data, each rotated by an angle 60[deg]≤φ≤120[deg] relative to the others. Denote the time of these measurements as $t_{A}$, $t_{B}$, and $t_{C}$.

The calibration process consists of two stages: first, obtaining a good initial guess using the measurements at $t_{A}$, $t_{B}$, and $t_{C}$; next, refining this initial estimate with all measurements for $t<t_{cal}$. The following subsections provide a detailed description of these steps.

To minimize potential inaccuracies of the calibration resulting from processing and communication delays during the parallel recording of the robot’s and the sensor’s orientations, the robot’s motion program should be designed carefully. Specifically, in the $t<t_{cal}$ interval, the end-effector should move at a low angular velocity and remain steady at $t_{A}$, $t_{B}$, and $t_{C}$ whenever possible.

### 3.1. Initial Guess

Consider the sensor pose at $t_{A}$, $t_{B}$ and $t_{C}$. The axis of rotation between two orientations gives a well-defined and easily obtainable common direction for both the sensor and the TCP.

For this, compute the direction of the axes as

(2) $$\left[t_{AB}^{\left(TCP\right)},\varphi_{AB}\right]==\mathrm{axis}\_\mathrm{angle}\left(q^{\left(base,TCP\right)}{\left(t_{A}\right)}^{-1}q^{\left(base,TCP\right)}\left(t_{B}\right)\right),$$

if the computation results in a negative $\varphi_{AB}$ angle, flip the direction of $t_{AB}^{\left(TCP\right)}$. (The computation requires that $\varphi_{AB}$, $\varphi_{BC}$, and the angles of $t_{AB}$ and $t_{BC}$ be as close to the right angle as possible.) Furthermore, vectors $t_{BC}^{\left(TCP\right)}$, $t_{AB}^{\left(sensor\right)}$ and $t_{BC}^{\left(sensor\right)}$ can be computed similarly.

Then, these values can be obtained by base and ref frames as

$$\begin{array}{cc} \; & t_{AB}^{\left(base\right)}=q^{\left(base,TCP\right)}\left(t_{B}\right)\cdot t_{AB}^{\left(TCP\right)},\\ \; & t_{AB}^{\left(ref\right)}=q^{\left(ref,sensor\right)}\left(t_{B}\right)\cdot t_{AB}^{\left(sensor\right)},\\ \; & t_{BC}^{\left(base\right)}=q^{\left(base,TCP\right)}\left(t_{B}\right)\cdot t_{BC}^{\left(TCP\right)},\\ \; & t_{BC}^{\left(ref\right)}=q^{\left(ref,sensor\right)}\left(t_{B}\right)\cdot t_{BC}^{\left(sensor\right)}. \end{array}$$

**Figure 3:**
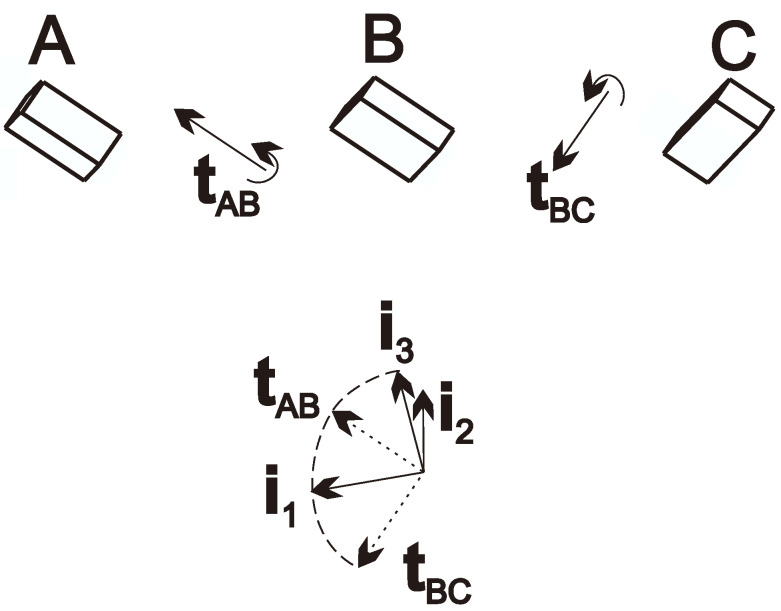
The sensor (illustrated by a box) in three (A, B, C) poses, the axes of rotation, and the resulted frame *i* with base vectors $i_{1}$, $i_{2}$, and $i_{3}$.

Now the $t_{AB}$ and $t_{BC}$ directions are known by all frames; the following orthogonal directions can be defined based on them:

(3) $$\begin{array}{cc} \; & i_{1}={\left(t_{AB}+t_{BC}\right)}_{norm},\\ \; & i_{2}={\left(t_{AB}\times t_{BC}\right)}_{norm},\\ \; & i_{3}=i_{1}\times i_{2}, \end{array}$$

which constructs a frame denoted by *i*. The method and the resulting frame are illustrated in Figure 3.

Determining these base vectors from the vectors given by the ref frame, the rotation matrix between the frames ref and *i* can be described as

(4) $$R^{\left(ref,i\right)}=\left[\begin{array}{ccc} i_{1} & i_{2} & i_{3} \end{array}\right].$$

It can also be computed for frames base, TCP and sensor as $R^{\left(base,i\right)}$, $R^{\left(TCP,i\right)}$ and $R^{\left(sensor,i\right)}$.

Then, the initial guess for quaternions $q^{\left(base,ref\right)}$ and $q^{\left(TCP,sensor\right)}$ can be computed as

(5) $$\begin{array}{cc} \; & q_{0}^{\left(base,ref\right)}=\mathrm{rotm}2\mathrm{quat}\left(R^{\left(base,i\right)}\cdot {\left(R^{\left(ref,i\right)}\right)}^{T}\right), \end{array}$$

(6) $$\begin{array}{cc} \; & q_{0}^{\left(TCP,sensor\right)}=\mathrm{rotm}2\mathrm{quat}\left(R^{\left(TCP,i\right)}\cdot {\left(R^{\left(sensor,i\right)}\right)}^{T}\right). \end{array}$$

### 3.2. Local Optimization

The previous step provided a good calibration for the three selected measurements. The local optimization step refines this calibration by taking into account all measurements for $0\leq t<t_{cal}$.

The correction of the orientations is defined with Roll–Pitch–Yaw variables (x)

(7) $$q_{RPY}\left(x\right)=\left(\cos\left(x_{1}/2\right)+i\sin\left(x_{1}/2\right)\right)\cdot \left(\cos\left(x_{2}/2\right)+j\sin\left(x_{2}/2\right)\right)\cdot \left(\cos\left(x_{3}/2\right)+k\sin\left(x_{3}/2\right)\right),$$

where small angles are assumed due to the good initial guess.

With this correction term, the quaternions can be defined as

(8) $$\begin{array}{cc} \; & q^{\left(base,ref\right)}\left(x\right)=q_{RPY}\left(x\right)\cdot q_{0}^{\left(base,ref\right)}, \end{array}$$

(9) $$\begin{array}{cc} \; & q^{\left(TCP,sensor\right)}\left(y\right)=q_{RPY}\left(y\right)\cdot q_{0}^{\left(TCP,sensor\right)}. \end{array}$$

According to the parameters x and y, the measurements can be evaluated as

$$\begin{array}{cc} \; & q^{\left(base,sensor\;by\;robot\right)}\left(t,x\right)=q^{\left(base,ref\right)}\left(x\right)q^{\left(ref,sensor\right)}\left(t\right),\\ \; & q^{\left(base,sensor\;by\;sensor\right)}\left(t,y\right)=q^{\left(base,TCP\right)}\left(t\right)q^{\left(TCP,sensor\right)}\left(y\right), \end{array}$$

then the error of the measurement can be computed as the angle of a quaternion

(10) $$\epsilon \left(t,x,y\right)=\mathrm{angle}(q^{\left(sensor\;by\;sensor,sensor\;by\;robot\right)}\left(t,x,y\right)),$$

where

$$q^{\left(sensor\;by\;sensor,sensor\;by\;robot\right)}\left(t,x,y\right)=q^{\left(base,sensor\;by\;sensor\right)}{\left(t,y\right)}^{-1}\cdot q^{\left(base,sensor\;by\;robot\right)}\left(t,x\right).$$

Based on this derivation, this optimization problem can be written as

(11) $$\begin{array}{c} \underset{x\in R^{3},y\in R^{3}}{\mathrm{minimize}}\sum_{t<t_{cal}}\epsilon {\left(t,x,y\right)}^{2}, \end{array}$$

where a Nelder–Mead optimization can be used initialized from x=0, y=0.

From the resulting x, y vectors, the error of a measurement can be written as

(12) ϵ(t)=ϵ(t,x,y).

## 4. Experimental Demonstration

This section presents examples of validating various orientation sensors using a UR16e manipulator [24] with the proposed method. The results provide a straightforward comparison of the devices’ performance. We investigated two sensors, a MEMS-based IMU sensor and the HTC VIVE VR headset (Manufacturer: TDK INVENSENSE (San Jose, CA, USA) Type ID: ICM-20948) equipped with 6DoF tracking capabilities (so-called inside-out tracking).

### 4.1. ICM20948 IMU with Disabled Magnetometer

The IMU sensor ICM20948 [25] used in the experiment integrates gyroscope, magnetometer, and accelerometer units in a single package. For this measurement, the magnetometer was turned off because of the disturbing magnetic field of the manipulator. It was found that the micro-vibrations of the manipulator can influence the performance of the sensor to a considerable extent causing noise and drift in the output signal. For this reason, the sensor was attached to the manipulator in silicon bedding instead of using a rigid mounting fixture.

**Figure 4:**
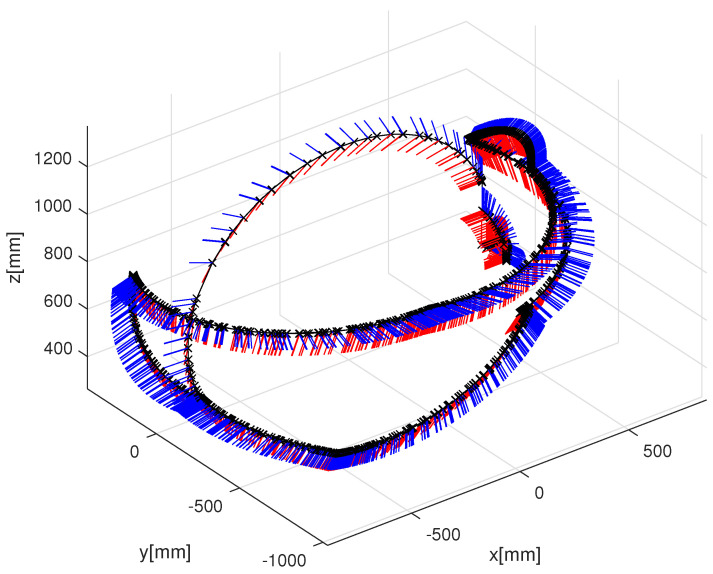
Three-Dimensional TCP path of the measurement. Red and blue segments show direction *x* and *z* respectively.

Furthermore, the effect of these vibrations was also diminished by applying a higher robot speed (2–4 [cm]/step). Figure 4 shows the path of the manipulator. After an initial motion phase, it performs five full circles. The figure also shows the orientation in each measurement point with red line segments in the *x* direction and blue segments in the *z* direction.

First, three measurements (samples recorded at $t_{A}$, $t_{B}$, and $t_{C}$) must be chosen as close to perpendicular as possible.

It was obtained by performing a search through the $t<t_{cal}$ section of the logged data, according to the following optimum criteria (see Section 3.1):

$$\begin{array}{cc} \underset{t_{A},t_{B},t_{C}<t_{cal}}{\mathrm{minimize}} & \max(|\delta_{AB}|,|\delta_{BC}|,|\delta_{t}|)\\ \mathrm{subject}\;\mathrm{to}\;\; & \delta_{AB}=\pi /2-\varphi_{AB},\\ \; & \delta_{BC}=\pi /2-\varphi_{BC},\\ \; & \delta_{t}=\pi /2-\mathrm{angle}\left(t_{AB},t_{BC}\right), \end{array}$$

where in optimal case $\delta =\max(|\delta_{AB}|,|\delta_{BC}|,|\delta_{t}|)=0$, so the angles are perpendicular.

On the initial phase of the path ($t<t_{cal}$), the procedure automatically looks for three measured orientations that are inclined at least 60 degrees and less than 120 degrees relative to each other (in this case, their indices were $i_{A}=1$, $i_{B}=374$ and $i_{C}=457$). The angle differences between the orientations are $\varphi_{AB}=89.65\;\left[\deg\right]$ and $\varphi_{BC}=89.29\;\left[\deg\right]$. Furthermore, the angle of $t_{AB}$ and $t_{BC}$ is 89.68[deg] because of the initial path segment, and δ is almost zero.

**Figure 5:**
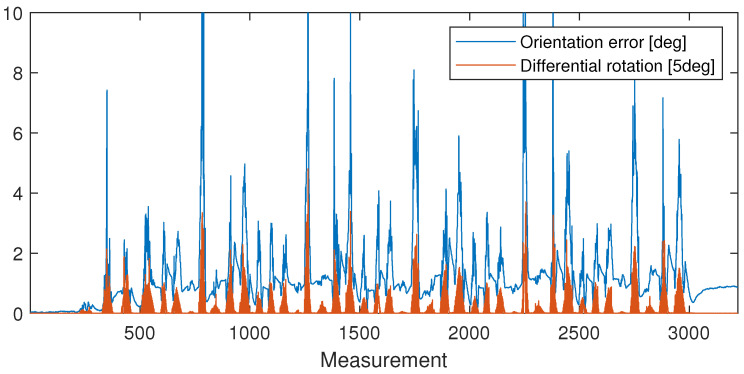
The computed ϵ(t) error of the IMU orientation according to the orientation computed from the robot model (blue); rotation angle between the measured TCP orientations (red). Larger errors occur only during transient motion.

**Figure 6:**
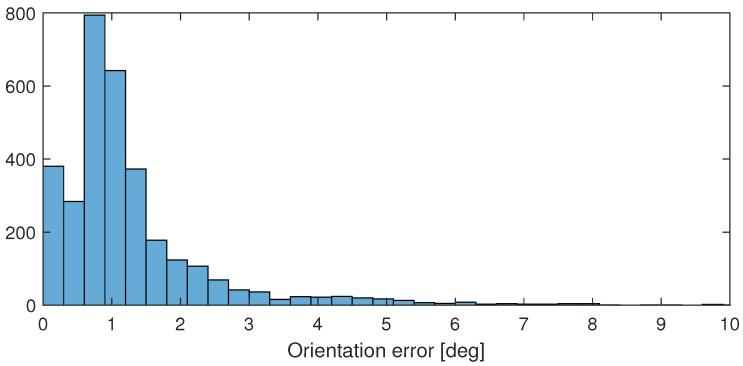
Histogram of the computed ϵ(t) error of the IMU orientation according to the orientation computed from the robot model.

With the results of calibration, the ϵ(t) error of the orientation of the IMU sensor (considering the robot as a reference) can be computed, and it is plotted in Figure 5 with blue. The histogram of the error is plotted in Figure 6. They clearly show that the error is mostly below 1.5[deg], and by plotting the magnitude of rotations between the measurements (in red in Figure 5), it is easy to see that significant errors are only measured during fast rotations. These errors are partially caused by the unavoidable time difference between signal processing in the robot and the sensors that result in a certain time delay between the parallel orientation samples.

It should be emphasized that the contribution of this paper is confined to the method and procedure for registering the robot’s and the orientation sensor’s references. Consequently, the investigation of the time delay is intentionally considered beyond the scope of this work. Measuring or identifying time delays in commercial systems (e.g., robot controllers; IMU’s internal signal processing, etc.), which are out of the researchers’ control, may not even be feasible in general. However, using the proposed procedure, a comparative delay analysis can be performed with multiple orientation sensors mounted on the robot simultaneously or consecutively. Such an analysis could be valuable from an engineering perspective when evaluating sensing technologies for specific applications.

### 4.2. HTC VIVE (IMU and SLAM Sensor-Fusion Using 6 Cameras)

The HTC VIVE Cosmos headset uses six cameras and an IMU unit for its SLAM algorithm and can be considered as a cutting-edge inside-out tracking technology. The headset was fixed to the flange of the manipulator and was moved on the path plotted in Figure 4, without the initial segment. Because it was not sensitive to vibrations of slow robot motions, two rounds were recorded at different speeds.

In this case, an automatic search was also applied to obtain orientation samples with 60[deg]≤φ≤120[deg] angles between them. The indices of the chosen measurements were $i_{A}=1$, $i_{B}=228$ and $i_{C}=1462$, and the angles between them are $\varphi_{AB}=30.03\;\left[\deg\right]$ and $\varphi_{BC}=36.29\;\left[\deg\right]$. Furthermore, the angle of $t_{AB}$ and $t_{BC}$ is 30.01[deg], so in this case, δ=60[deg].

**Figure 7:**
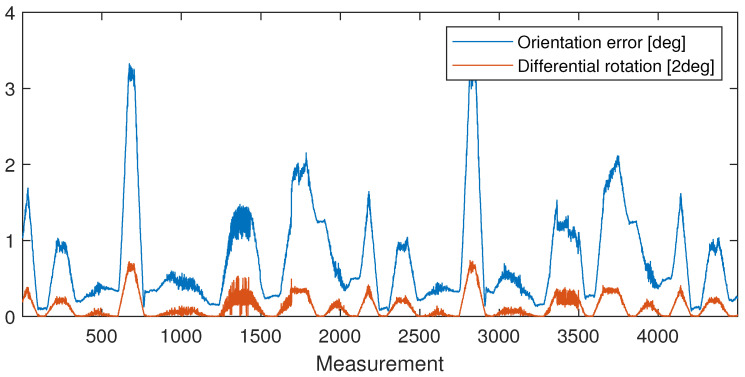
The computed ϵ(t) error of the HTC VIVE orientation according to the orientation computed from the robot model (blue); rotation angle between the measured TCP orientations (red). Larger errors occur only during transient motion.

**Figure 8:**
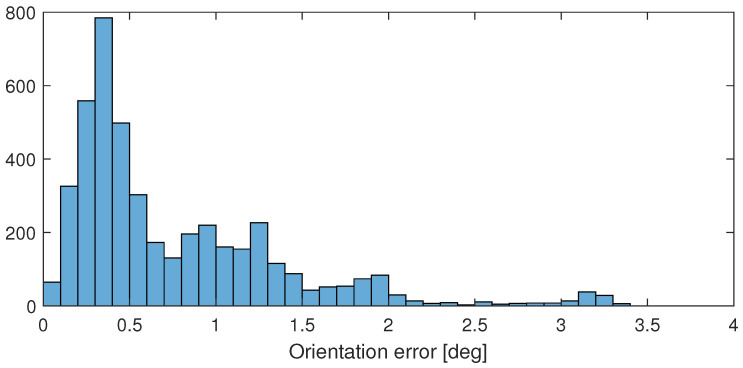
Histogram of the computed ϵ(t) error of the HTC VIVE orientation with respect to the orientation computed from the robot model.

After performing the calibration method described in Section 3, the computation results in ϵ(t) errors plotted in Figure 7 in blue and in the histogram of Figure 8. The figures show that the error is mostly below 0.6[deg]. The rotation angle between the measurements is also plotted in Figure 7 with red, and it clearly shows that the larger differences between the sensors are measured only if the device is rotating.

### 4.3. Notes on the Robot Trajectory

The proposed method is designed to be robust to the robot’s motion during measurement, provided that the key poses required for calibration are carefully selected (See Section 3). Specifically, the robot path must be planned to avoid kinematic singularities, as approaching these configurations can lead to large joint velocities, causing vibrations and potentially affecting the accuracy of orientation measurements.

Outside of these singularities, the robot’s path has no impact on the algorithm’s sensitivity, since the calibration process depends solely on the relative angular separation between the key poses. As long as these key poses are properly captured during the calibration phase and the relative orientation between the robot’s end-effector and the sensor is preserved, the method will maintain its reliability. By ensuring smooth, controlled robot motions and steering clear of singular configurations, the robustness of the proposed method can be upheld across various trajectories.

## 5. Future Work

Concerning further research, two directions are considered. The first goal is performing experimental work on the validation of standalone 6DoF tracking sensors, like [30], on a similar robotic setup, extending the investigation to positioning accuracy. The other research direction aims to apply the presented approach for the geometric calibration of manipulators, as well as the real-time monitoring of the consistency between the joint trajectory and the Cartesian trajectory of robot manipulators with a known kinematic model. Such a monitoring method can be beneficially applied in mission-critical medical robotic systems, e.g., the CyberKnife [31].

## 6. Conclusions

This paper proposed a generic method to validate orientation sensors using an independent reference of higher accuracy. The method has two main steps. First, a good initial guess is computed via simple geometric computation, which is followed by a refinement via local optimization starting from the previously computed initial guess. The method is presented in a generic manner, enabling its application with any kind of programmable manipulator and the associated orientation measurement instrumentation. Commercial robot arms are compact and reliable devices suitable for this purpose. The main benefits of the proposed method include the repeatable excitation pattern that allows for the comparison of various orientation sensors under the same conditions regarding the orientation trajectory, external disturbances, visual environment, etc. The main limitation of the method is its reliance on the assumption that the robot’s orientation measurements are perfectly accurate. Furthermore, the method may encounter challenges when applied to sensors with significant drift, as this can lead to larger discrepancies during the calibration process. Nevertheless, these limitations are generally not critical for most engineering applications. The method is illustrated by validating an IMU (ICM20948) and a complex inside-out tracking system (HTC VIVE Cosmos) with a UR16e manipulator. The experiments show that concerning the investigated sensors, significant orientation errors only occur at larger angular velocities.

## Data Availability

Measurement data and supplementary photo documentation is available at: https://github.com/ABC-iRobotics/OrientationSensorValidatinon-PublicData-MDPI-Sensors (accessed on 20 October 2024).

## Appendix A

The orientations is described via unit quaternions. The main operations for their usage are discussed here. Denote the unit quaternion as

q=w+xi+yj+zk,

where $w^{2}+x^{2}+y^{2}+z^{2}=1$. The related rotation matrix can be written as

$$R_{q}=\left[\begin{array}{ccc} 1-2(y^{2}+z^{2}) & 2xy-2zw & 2xz+2yw\\ 2xy+2zw & 1-2(x^{2}+z^{2}) & 2zy-2x\;w\\ 2xz-2yw & 2yz+2xw & 1-2(x^{2}+y^{2}) \end{array}\right].$$

The quaternion form a rotation matrix can be computed via the Stephenson formula; see [32]. The quaternion of a rotated orientation around axis t by angle φ is

$$q=\cos\left(\varphi /2\right)+\sin\left(\phi /2\right)\left(t_{x}i+t_{y}j+t_{z}k\right),$$

and its opposite too. The angle from a quaternion can be computed as

$$\varphi =2\cdot atan2(\sqrt{x_{3}^{2}+y_{3}^{2}+z_{3}^{2}},w_{3}),$$

and the axis from the direction of the complex part, taking into account the sign of the real part.

The rotation of a vector v by a q quaternion (q·v) can be computed as $R_{q}\cdot v$ matrix product.

If a vector represents a v direction described by frame A, it is written as $v^{\left(A\right)}$.

Furthermore, the indexing of rotation matrices and quaternions must be understood as

$$\begin{array}{cc} \; & v^{\left(B\right)}=q^{\left(B,A\right)}\cdot v^{\left(A\right)},\;\mathrm{and}\\ \; & v^{\left(B\right)}=R^{\left(B,A\right)}\cdot v^{\left(A\right)}. \end{array}$$

The quaternion that represents the inverse rotation can be computed as

$$q^{-1}=-w+xi+yj+zk.$$

Denote another unit quaternion as

$$q_{2}=w_{2}+x_{2}i+y_{2}j+z_{2}k,$$

The product of rotations q and $q_{2}$ for computations like $q\cdot (q_{2}\cdot v)$ can be computed as

$$\begin{array}{c} q\cdot q_{2}=ww_{2}-xx_{2}-yy_{2}-zz_{2}+i\left(wx_{2}+xw_{2}-yz_{2}+zy_{2}\right)\\ +j\left(wy_{2}+xz_{2}+yw_{2}-zx_{2}\right)+k\left(wz_{2}-xy_{2}+yx_{2}+zw_{2}\right). \end{array}$$

The angle of orientations A and B can be computed as the angle of quaternion $q^{\left(A,B\right)}$.

The normalization of a vector is denoted as

$${\left(v\right)}_{norm}=v/||v||.$$

The angle of two vectors v, $v_{2}$ is computed as

$$\varphi =\mathrm{acos}(v^{T}v_{2}/\left(||v|\right|\cdot \left|\right|v_{2}||)).$$
